# Reliability and validity of the DEFISS score for predicting post-extubation dysphagia and dysphagia-related reintubation after stroke

**DOI:** 10.1038/s41598-026-62133-x

**Published:** 2026-07-17

**Authors:** Paul Muhle, Niklas Weßeler, Anne Jung, Rainer Dziewas, Bendix Labeit, Antje Schmidt-Pogoda, Oliver Grauer, Sonja Suntrup-Krueger

**Affiliations:** 1 Department of Neurology, University Hospital Muenster, Building A1, Albert-Schweitzer-Campus 1, 48149 Muenster, Germany; 2 Department of Anesthesiology, Intensive Care and Pain Medicine, University Hospital Muenster, Muenster, Germany; 3 Department of Neurology, Klinikum Osnabrück, Osnabrück, Germany; 4 Department of Neurology, University Hospital Duesseldorf, Düsseldorf, Germany

**Keywords:** Dysphagia, Stroke, Extubation failure, Interrater reliability, FEES, FEDSS, Intensive care, Diseases, Health care, Medical research, Neurology

## Abstract

Post-extubation dysphagia is frequent after severe stroke and can lead to pneumonia, delayed recovery, and dysphagia-related reintubation. We evaluated the Determine Extubation Failure in Severe Stroke (DEFISS) score, a bedside tool combining stroke-specific clinical factors, duration of mechanical ventilation, and a brief oral motor function assessment to support pre-extubation risk stratification. In a prospective single-center cohort, three blinded raters (novice, intermediate, expert) independently scored DEFISS immediately before planned extubation, which followed routine clinical care. After extubation, all patients underwent flexible endoscopic evaluation of swallowing by assessors blinded to DEFISS. The primary outcome was dysphagia-related reintubation within 120 h, and construct validity was assessed against the Fiberoptic Endoscopic Dysphagia Severity Scale. Thirty-nine stroke patients (mean age 73.9 ± 11.6 years; 54% female) were included; five (12.8%) experienced dysphagia-related reintubation. Interrater reliability was excellent (ICC_single 0.887; ICC_average 0.959), with similarly high reliability for the oral motor function component (ICC_single 0.874). DEFISS correlated with endoscopic dysphagia severity (ρ = 0.44, *p* = 0.005). Using a prespecified cutoff (DEFISS ≥ 4), odds of dysphagia-related reintubation increased (OR 11.1, *p* = 0.035) with sensitivity 0.80 and specificity 0.73. DEFISS may provide a pragmatic triage tool to prioritize early FEES and targeted dysphagia management in critically ill stroke patients.

**Supplementary Information:**

The online version contains supplementary material available at 10.1038/s41598-026-62133-x.

## Introduction

Post-extubation dysphagia (PED) is increasingly recognized as a major complication in critically ill patients, with reported incidences ranging from 12% to 60% depending on population, methods, and timing of assessment^1–5^. Stroke patients are particularly vulnerable, reflecting the interplay of structural brain injury, impaired consciousness, and prolonged mechanical ventilation^6,7^. The mechanisms underlying PED are multifactorial: neurogenic dysphagia results from cortical and subcortical lesions, brainstem involvement, and impaired sensory-motor integration^1,8–10^, while ICU-acquired weakness and iatrogenic factors such as intubation-related laryngeal trauma and reduced pharyngeal sensation further exacerbate swallowing dysfunction^11–14^.

Among stroke patients, dysphagia has emerged as the predominant determinant of extubation failure, outweighing conventional respiratory and hemodynamic indices^15,16^. Severe dysphagia documented by Flexible Endoscopic Evaluation of Swallowing (FEES) after extubation has proven to be the single strongest predictor of reintubation^15^. This finding underscores that, in neurocritical patients, airway protection failure – rather than ventilatory insufficiency – is the primary driver of extubation outcomes. Reintubation after stroke is associated with a cascade of adverse consequences, including aspiration pneumonia, prolonged mechanical ventilation and stay in the ICU, tracheostomy, and higher mortality^17–22^. Each failed extubation increases ICU length of stay by up to one week and doubles in-hospital death risk in neurological populations^21^. Determining the right time for safe extubation and minimizing the risk of extubation failure through swallowing function assessment is therefore a key target in stroke airway management.

Despite this, bedside dysphagia screening practices in Neuro-ICUs remain heterogeneous. The international „Dysphagia in Intensive Care Evaluation“ (DICE) survey highlighted wide variability in timing, screening responsibility, and use of instrumental assessments across units, underscoring the need for structured, implementable tools at the bedside^23^. Existing stroke-unit screening instruments (e.g., Gugging Swallowing Screen, Volume–Viscosity Swallow Test, two-minute test) perform well for aspiration risk in non-intubated patients^7,24–28^, but they were not designed for the immediate pre-extubation setting. In the ICU, the GUSS-ICU^29^ has been validated for general extubated populations, yet stroke-specific pre-extubation tools to assess airway protective capacity are lacking. Instrumental approaches such as FEES-based dysphagia severity grading (e.g., Fiberoptic Endoscopic Dysphagia Severity Scale [FEDSS]) offer excellent diagnostic accuracy^30,31^, but are not feasible in intubated patients. Likewise, structured endoscopic protocols for tracheostomy decannulation, such as the Standardized Endoscopic Swallowing Evaluation for Tracheostomy Decannulation (SESETD)^32–34^, have demonstrated high reliability and validity but cannot be applied before extubation.

To address this gap, the Determine Extubation Failure in Severe Stroke (DEFISS) score was developed as a pragmatic pre-extubation risk stratification tool. DEFISS combines stroke-specific clinical factors with a focused oral motor function (OMF) assessment^15,35^ to identify stroke patients at risk of dysphagia-related extubation failure. While early work reported promising diagnostic performance of DEFISS, independent prospective replication with a formal focus on interrater reliability, blinded assessment, and bedside implementability across experience levels is required to support routine clinical uptake.

## Methods

### Design and setting

We conducted a prospective observational study in a neurological ICU at a tertiary academic center in Germany at University Hospital Muenster. Consecutive adult stroke patients between May 2023 and August 2025 undergoing planned extubation within the next three hours from bedside evaluation were included. DEFISS was scored by three independent raters and scores were not disclosed to treating teams and did not influence extubation decisions. Patients with non‑stroke primary diagnoses were excluded from the primary analysis but assessed exploratively for OMF (Supplement).

### Raters and procedures

Raters received a brief standardized instruction. Experience strata were predefined according to ICU experience (novice < 2 years, intermediate 2–5 years, expert > 5 years). This classification was intentionally chosen to reflect real-world bedside extubation practice, where clinicians with varying levels of ICU experience commonly perform pre-extubation assessments. DEFISS was designed as a pragmatic bedside tool and can be completed at the bedside in approximately 3–5 min. DEFISS comprises four components: (i) OMF bedside score (0–11 points; Supplement), (ii) duration of mechanical ventilation (≥ 24 h vs. < 24 h), (iii) lesion location (supratentorial vs. infratentorial), and (iv) stroke severity (NIHSS categories < 5, 5–15, > 15). The four DEFISS components were originally derived in a prior prospective cohort study of mechanically ventilated stroke patients^15^, in which multiple candidate pre-extubation variables were entered into multivariable regression models. Duration of ventilation, oral motor function, infratentorial lesion location, and stroke severity emerged as the independent pre-extubation predictors of extubation failure and were therefore incorporated into the score. Details and scoring rules have been published elsewhere^15^ and are provided in additional file 1. The instruction consisted of a 20 min structured verbal training session, a one-page written scoring sheet with item anchors, and discussion of two example cases not included in the study cohort.

Based on pilot observations and clinical plausibility, a DEFISS ≥ 4 cutoff was prespecified to maximize bedside rule-out capability for dysphagia-related extubation failure^15^. All raters were mutually blinded and blinded to outcomes; patients were clinically weaned according to ICU standards prior to DEFISS scoring. The instrument does not assess ventilatory mechanics or gas exchange and must be interpreted in addition to respiratory readiness criteria^15,36,37^. All patients underwent FEES within 24 h after extubation, and dysphagia severity was graded using the FEDSS, a validated instrument specifically developed for the acute stroke setting^30,31^. The FEDSS grades swallowing safety on a six-point ordinal scale, ranging from normal function (1 pt.) to saliva aspiration (6 pts.), and has been shown to predict the risk of post-stroke pneumonia and adverse functional outcomes. The FEES assessors were blinded to DEFISS. In the present study, the FEDSS served as a reference standard for dysphagia assessment and was used to evaluate the construct validity of the DEFISS through correlation analysis. This approach allowed us to benchmark the bedside-derived DEFISS against an established, FEES-based gold-standard measure of swallowing function.

### Outcomes and variables

The primary outcome was dysphagia-related extubation failure, defined a priori as reintubation within 120 h for airway protection failure due to dysphagia. Causes of reintubation were independently adjudicated from source records by two investigators blinded to DEFISS and categorized as dysphagia-related vs. non-dysphagia related; disagreements were resolved by consensus. Secondary outcomes were post-extubation pneumonia, ICU length of stay, post-extubation swallowing function, and in-hospital mortality. Baseline variables included age, sex, lesion location, stroke severity (National Institutes of Health Stroke Scale, NIHSS), duration of mechanical ventilation, ventilation parameters and interventions (thrombolysis, thrombectomy, external ventricular drainage, decompressive surgery).

### Statistical analysis

Interrater reliability was quantified using two-way mixed-effects Intraclass Correlation Coefficient (ICC; absolute agreement), reported for single- and average measures with 95% Confidence Intervals (CI). Item-level OMF ICCs were calculated analogously. Two-way mixed, absolute-agreement ICCs were chosen to reflect fixed raters and clinical need for exact score reproducibility. Construct validity was evaluated by Spearman correlation between DEFISS (consensus) and FEDSS. Predictive validity was assessed for (i) DEFISS as a continuous predictor (Receiver Operating Curve [ROC] Area under the Curve [AUC] with 95% CI) and (ii) the pre-specified threshold (≥ 4) with 2 × 2 metrics (sensitivity, specificity, positive predictive value [PPV], negative predictive value [NPV] with exact binomial 95% CIs), odds ratio (Fisher’s exact), and calibration (Brier score). Sensitivity analyses included (a) using the novice rater’s DEFISS only and (b) averaging the three raters. ROC analyses used the nonparametric AUC with asymptotic SE and 95% CI as implemented in SPSS; ties between positive and negative cases were present. Where asymptotic upper bounds exceeded 1.000, CIs were truncated at 1.000. Sensitivity analyses included (A) novice-only and (B) averaged-rater thresholds (Table S2). Full ROC coordinates and Youden’s J are provided (see additional file 1: Table S1). Analyses used complete-case data; variable-wise missingness was < 10% and not imputed. Two-sided *p* < 0.05 indicated statistical significance. Reporting follows GRRAS (Guidelines for Reporting Reliability and Agreement Studies) for reliability analyses and STROBE (Strengthening the reporting of observational studies in epidemiology) for observational cohort design. Key design choices (blinding, rater training, handling of missing data) are detailed below; full GRRAS/STROBE checklists are provided (see Supplementary Information: STROBE and GRRAS checklists). Analyses were performed using SPSS version 29.0 (IBM Corp., Armonk, NY, USA).

### Use of large language models

Drafting and language polishing were supported by a large language model (ChatGPT 5.0). All content was critically reviewed, verified for accuracy, and approved by the authors, who take full responsibility for the work.

### Ethics approval and consent to participate

This study was approved by the local ethics committee of the Medical Faculty, University of Münster (reference number: 2023-234-f-S). Written informed consent was obtained from all participants or their legal representatives in accordance with national regulations and the Declaration of Helsinki.

## Results

### Cohort

**Fig. 1:**
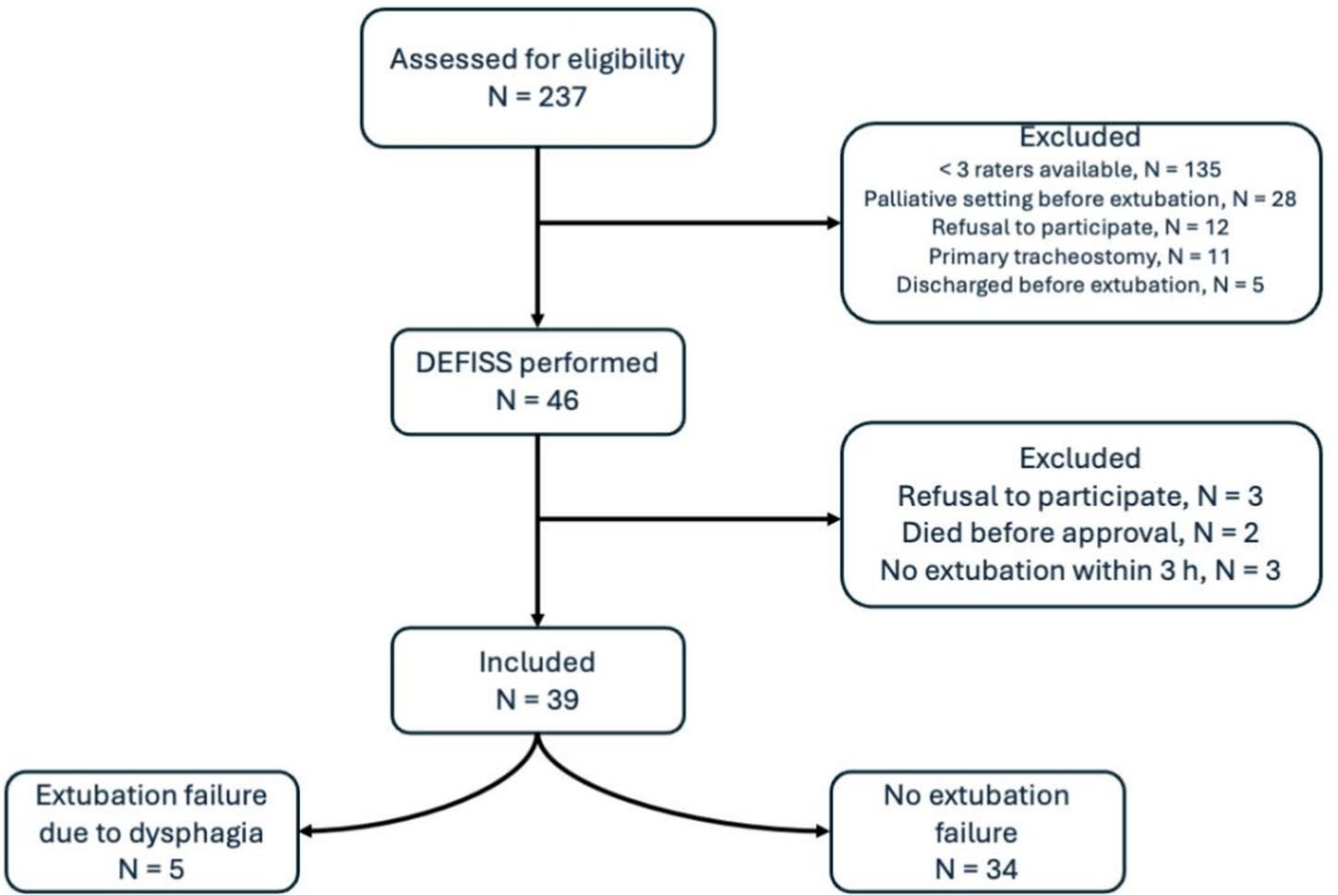
Study flow diagram. Screening, exclusions, and final numbers included in reliability and validity analyses (stroke cohort).

Thirty‑nine ventilated stroke patients were included (mean age 73.9 ± 11.6 years; 54% female). Baseline characteristics are shown in Table 1. Five patients (12.8%) required reintubation due to dysphagia within 120 h; overall, reintubations were eight (20.5%). FEES was performed at 9.9 ± 11.9 h post-extubation. Patient flow is shown in Fig. 1.

### Interrater reliability

DEFISS showed excellent agreement (ICC_single_ = 0.887, 95% CI 0.818–0.935; ICC_average_ = 0.959, 95% CI 0.931–0.977; see Tables 2 and 3). The OMF subscore was similarly robust (ICC_single_ = 0.874). For the binary risk class (DEFISS ≥ 4), agreement was substantial to excellent: Cohen’s κ (novice vs. expert) = 0.83 (Standard Error [SE] 0.09; *p* < 0.001) and Fleiss’ κ (three raters) = 0.77 (observed 0.90; expected 0.56).

### Construct validity and sensitivity analyses

The DEFISS final (consensus) score correlated with post‑extubation FEDSS (Spearman’s ρ = 0.44, *p* = 0.005; see Table 4), supporting convergence between pre‑extubation bedside OMF impairment and endoscopic dysphagia severity. The Brier score for the DEFISS prediction model was 0.068 (SD 0.21), indicating good overall calibration. Given the cohort event rate (12.8%), values ≪0.112 (null model) indicate better-than-baseline calibration for the pre-specified threshold prediction. Using only the novice rater’s DEFISS yielded similar discrimination and threshold performance; averaging the results of three raters did not materially change effect estimates (Table S2).

**Table 1:** Baseline characteristics of the stroke cohort (*n* = 39). Values are mean ± Standard Deviation (SD) or n (%), as indicated. Abbreviations: GCS, Glasgow Coma Scale; NIHSS, National Institutes of Health Stroke Scale; PACS, partial anterior circulation stroke; POCS, posterior circulation stroke; ICH, intracerebral hemorrhage; FEES, flexible endoscopic evaluation of swallowing; FEDSS, Fiberoptic Endoscopic Dysphagia Severity Scale; ICU, intensive care unit; pts., Points; d, days; ASB = assisted spontaneous breathing; PEEP, positive endexpiratory pressure; DEFISS, Determine Extubation Failure in Severe Stroke; OMF, Oral Motor Function (Scale);

Characteristic	Value
Age, mean ± SD (years)	73.95 ± 11.63
Female sex, n (%)	21 (53.8)
Primary diagnosis	
Supratentorial stroke	29 (76.9%)
PACS / POCS	2 (5.1%) / 1 (2.6%)
ICH	4 (10.3%)
Infratentorial stroke	2 (5.1%)
Multilocular	1 (2.6%)
Lesion side	Left: 21 (53.8%); Right: 13 (33.3%); Bilateral: 4 (10.3%)
NIHSS at admission, mean ± SD (pts.)	12.74 ± 6.34
Systemic thrombolysis, n (%)	17 (43.6)
Thrombectomy / stenting, n (%)	30 (76.9)
Hemicraniectomy, n (%)	6 (15.4)
External ventricular drainage, n (%)	9 (23.1)
GCS before extubation (pts.)	12–13: 7 9–11: 30 7–8: 2
FiO2 before extubation (%), mean ± SD	0.28 ± 0.06
ASB before extubation (Δmbar), mean ± SD	3.46 ± 2.83
PEEP before extubation (mbar), mean ± SD	5.21 ± 0.66
Respiratory rate before extubation, mean ± SD (min⁻¹)	17.61 ± 3.42
Tidal volume (mL) before extubation, mean ± SD	477.44 ± 116.16
DEFISS, mean ± SD	2.62 ± 1.55
OMF, mean ± SD	2.99 ± 2.08
Duration of mechanical ventilation, mean ± SD (h)	59.88 ± 58.75
Pneumonia after extubation, n (%)	22 (56.4)
Time from extubation to FEES (h)	9.89 ± 11.90
FEDSS after extubation, mean ± SD	4.26 ± 1.35
Length of stay, mean ± SD (d)	14.15 ± 9.16
Tracheostomy during admission, n (%)	4 (10.3)
In‑hospital mortality, n (%)	3 (7.7)
Reason for initial intubation, n (%)	Intervention: 35 (89.7) Poor oxygenation/absent reflexes: 4 (10.3)
Reintubation, n (%)	8 (20.5)
Reason for reintubation, n (%)	Dysphagia: 5 (12.8) Secondary intervention/complication: 2 (5.1) Vocal cord palsy: 1 (2.6)

**Table 2:** Interrater reliability (Intraclass Correlation Coefficient = ICC) for DEFISS (Determine Extubation Failure in Severe Stroke) and OMF (Oral Motor Function) in stroke (*n* = 39). Single- and average-measures ICCs with 95% CIs (Confidence Interval).

Score	ICC (Single)	95% CI	ICC (average measures)	95% CI
DEFISS total	0.887	0.818–0.935	0.959	0.931–0.977
OMF total	0.874	0.798–0.927	0.954	0.922–0.974

**Table 3:** Item-level interrater reliability (Intraclass Correlation Coefficient = ICC) for Oral Motor Function (OMF) in stroke (*n* = 39). Single- and average-measures ICCs with 95% CIs (Confidence Interval).

OMF item	ICC (Single)	95% CI	ICC (Average)	95% CI
Saliva management	0.741	0.606–0.844	0.896	0.822–0.942
Swallowing	0.799	0.688–0.880	0.922	0.868–0.957
Lip closure	0.648	0.487–0.782	0.846	0.737–0.914
Tongue motility	0.718	0.575–0.828	0.884	0.803–0.935
Jaw motility	0.597	0.425–0.744	0.816	0.689–0.897

### Predictive validity

Analyses were based on *n* = 39 with five dysphagia-related reintubations; three additional reintubations were non-dysphagia-related (secondary intervention/complication *n* = 2; vocal cord palsy *n* = 1). Using DEFISS ≥ 4, odds of dysphagia-related reintubation were higher (OR 11.1; *p* = 0.035) with sensitivity 0.80 (4/5), specificity 0.73 (25/34), PPV 0.31 (4/13), NPV 0.96 (25/26), accuracy 0.74 (29/39), LR + 2.96, and LR − 0.27. As a continuous predictor, AUC was 0.744 (SE 0.142; 95% CI 0.466–1.000; *p* = 0.085). The Youden-optimal cutoff was ≈ 3.33 (J = 0.506), broadly consistent with the prespecified ≥ 4 threshold (full ROC coordinates in Table S3).

In 11 non‑stroke ICU patients, OMF reliability between raters was excellent (ICC_average_ 0.92) but showed poor association with reintubation failure (see Supplementary File 1).

**Table 4:** Validity metrics for DEFISS against extubation failure in stroke (*n* = 39). Spearman correlation with FEDSS; ROC AUC for continuous DEFISS; and diagnostic performance of the pre-specified threshold (DEFISS ≥ 4).

Metric	Value	95% CI / *p*	Notes
Spearman’s ρ (DEFISS vs. FEDSS)	0.44	*p* = 0.005	*n* = 38–39
AUC (continuous DEFISS)	0.74	0.466–1.000 *p* = 0.085	ROC analysis
Cutoff ≥ 4: sensitivity	0.80	-	TP 4 / FN 1
Cutoff ≥ 4: specificity	0.73	-	TN 25 / FP 9
Cutoff ≥ 4: odds ratio	11.1	95% CI 1.09–113.1; *p* = 0.035	Fisher’s exact

## Discussion

We demonstrate that DEFISS can be scored reproducibly at the bedside across experience levels, with ICCs in the excellent range for the total score and OMF subscore, and only minor variability across OMF items. Item-level analysis revealed slightly lower agreement for jaw and lip motility; however, this did not materially affect total-score reliability. Construct validity was supported by the correlation with FEES-based FEDSS (ρ = 0.44), and the pre-specified cutoff (≥ 4) identified patients at substantially higher risk of dysphagia-related reintubation (OR ≈ 11). Despite limited number of dysphagia-related events, the prespecified DEFISS threshold (≥ 4) identified a clinically meaningful high-risk subset while maintaining a high negative predictive value (0.96), supporting pragmatic rule-out use in weaning-ready stroke patients. The continuous AUC was moderate (0.744) with wide CIs, reflecting low event rates; nonetheless, fixed-threshold performance remained operationally useful for triage.

While stroke-specific bedside screens have proven valuable in stroke units, their design does not address the unique needs of intubated ICU patients. The GUSS-ICU^29^ represents an important step for general ICU populations but lacks stroke-specificity. FEES-based assessments such as FEDSS provide detailed visualization and high diagnostic accuracy, yet their implementation immediately before extubation is limited by feasibility. Against this background, DEFISS fills an unmet need by integrating stroke-related clinical predictors with OMF testing into a rapid, stroke-specific screening instrument. Importantly, conventional respiratory indices such as rapid shallow breathing or oxygenation thresholds are insufficient to predict extubation success in brain-injured patients. Instead, impaired airway protection, reduced consciousness, and dysphagia drive outcomes^38^. Large-scale data from the „Extubation in neurocritical care patients“ (ENIO) study confirmed that neurological dysfunction is an independent predictor of extubation failure^21^. By explicitly integrating stroke-related risk factors and OMF, DEFISS operationalizes this conceptual shift, providing a structured bedside alternative to purely respiratory-focused criteria. Implementation surveys indicate substantial variability in who screens, when screening occurs post-extubation, and when to escalate to FEES in ICUs^23,39^. By requiring minimal training and no instrumentation, DEFISS could standardize a pre-extubation checkpoint and create a common triage language between intensivists, neurologists, and Speech and Language Therapy (SLT) teams.

Our findings also complement structured approaches developed for tracheostomy decision-making. The Stroke-related Early Tracheostomy Score (SETscore) predicts early tracheostomy based on neurological and airway parameters^40^, whereas the recent SETPOINT2 trial demonstrated that early tracheostomy does not improve functional outcome compared with a later, selective approach^41^. Within a unified airway strategy, high DEFISS scores may justify early, post-extubation FEES and contingency planning (including tracheostomy discussion if ventilator liberation is otherwise achieved). In contrast, low scores may support consideration of extubation within a broader clinical assessment – consistent with selective, later tracheostomy strategies.

In our cohort, more than half of patients developed pneumonia, and one in five required reintubation, consistent with prior observations^21,36,37^. While only five cases were directly attributable to dysphagia, these findings underline the vulnerability of this population. The high negative predictive value of DEFISS (0.96) suggests that it may be instrumental as a rule-out tool to identify low-risk patients suitable for extubation. Conversely, high scores should prompt early instrumental assessment and multidisciplinary planning. Nevertheless, the heterogeneity of reintubation causes – such as surgical re-intervention, bleeding, or vocal cord palsy – highlights that DEFISS must always be interpreted in conjunction with comprehensive clinical evaluation. In practice, DEFISS < 4 may indicate a lower-risk constellation, whereas DEFISS ≥ 4 may justify closer monitoring, early FEES (ideally within the first post-extubation hours), and multidisciplinary planning after extubation, including early SLT involvement and consideration of therapy (e.g., Pharyngeal Electrical Stimulation [PES]), where available. The combination of moderate AUC and high NPV aligns with a pragmatic bedside use case: DEFISS may support post-extubation risk stratification and prioritization of early FEES/SLT assessment and enhanced monitoring in patients with higher scores.

The strong inter-rater reliability observed for the OMF subscore aligns with previous evidence that simple clinical tasks, when standardized, can achieve reproducibility comparable to complex endoscopic assessments^34,42^. Crucially, DEFISS can be completed within 3–5 min and does not require specialized equipment, underscoring its feasibility for routine use at the bedside. This efficiency distinguishes it from endoscopic protocols, which, although highly informative, are resource-intensive and not universally available.

DEFISS has already been operationalized as a stratification tool in interventional work: in a recent pilot study, high-risk patients (by DEFISS) received PES prior to extubation with signals toward improved swallowing and fewer reintubations. This underscores DEFISS’s potential not only for prognostication but also for targeting resource-intensive interventions (early FEES, SLT, PES) to those most likely to benefit^43^. The ability to stratify patients pre-extubation aligns with emerging therapeutic strategies: early initiation of speech and language therapy has been associated with improved outcomes in PED^44^, and structured oral care and swallowing interventions likewise show potential benefits in reducing complications^45^. Moreover, neuromodulation techniques such as PES are increasingly investigated, with accumulating evidence supporting their feasibility and potential to reduce extubation failure rates^43,46–49^. DEFISS may thus serve as a triage tool to identify those most likely to benefit from such targeted interventions, as shown in a pilot-trial by our group^43^.

This single-centre design with standardized weaning and early FEES may limit generalizability, and external validation across different ICU settings will be necessary. Although raters and FEES assessors were blinded, downstream care could have been influenced by clinical impressions beyond DEFISS. Several factors may have influenced both DEFISS scoring and extubation outcomes. Reduced vigilance, residual sedation, or delirium may transiently worsen cooperation and oral motor task performance, thereby increasing DEFISS despite potentially reversible impairment. ICU-acquired weakness or secretion burden may compromise cough effectiveness and airway clearance, increasing reintubation risk independent of primary swallowing function. Likewise, intubation-related laryngeal dysfunction may impair airway protection despite otherwise acceptable respiratory readiness. Accordingly, DEFISS should be interpreted as an adjunct to comprehensive clinical assessment. Only five reintubations were adjudicated as dysphagia-related, limiting statistical power and precision of predictive estimates. Confidence intervals were wide, and threshold performance should therefore be interpreted cautiously. Larger multicenter cohorts are required for robust external validation, calibration, and possible refinement of score weighting. In addition, rater expertise was operationalized by ICU experience rather than by formal dysphagia-specific qualifications or documented assessment volume; although this reflects real-world extubation practice, it may not capture all dimensions of extubation-related expertise. Although the score items were derived from a prior prospective cohort, future larger datasets may allow refinement of weighting and evaluation of additional candidate predictors. Long-term swallowing and functional outcomes were not assessed.

## Conclusion

In summary, DEFISS represents the first stroke-specific bedside screening tool for reintubation risk prior to extubation. It combines high interrater reliability with feasibility and clinical relevance. By bridging the gap between generic ICU screens and resource-intensive FEES assessments, DEFISS may facilitate timely identification of high-risk patients and support extubation risk stratification in stroke patients.

## Supplementary Information

Below is the link to the electronic supplementary material.

Supplementary Material 1

Supplementary Material 2

## Data Availability

The datasets generated and analyzed during the current study are available from the corresponding author on reasonable request.

## Abbreviations

AUC: Area under the receiver operating characteristic curve
CI: Confidence interval
DEFISS: Determine Extubation Failure in Severe Stroke
FEES: Flexible Endoscopic Evaluation of Swallowing
FEDSS: Fiberoptic Endoscopic Dysphagia Severity Scale
ICC: Intraclass correlation coefficient
ICU: Intensive Care Unit
LR+: Positive likelihood ratio
LR−: Negative likelihood ratio
NPV: Negative predictive value
OMF: Oral Motor Function
OR: Odds ratio
PED: Post–extubation dysphagia
PPV: Positive predictive value
ROC: Receiver operating characteristic
SE: Standard error
SLT: Speech and Language Therapy
