# Supplementary material for: Reliability and validity of the DEFISS score for predicting post-extubation dysphagia and dysphagia-related reintubation after stroke
Source: Sci Rep. 2026 Jul 17;16:22536. doi: 10.1038/s41598-026-62133-x (PMC13379578; doi:10.1038/s41598-026-62133-x)
Supplement: Supplementary file 1 — Supplementary Material 1 [file 41598_2026_62133_MOESM1_ESM.docx]

# Supplementary material

# Exploratory analysis in non-stroke patients

A total of 11 non-stroke patients who underwent mechanical ventilation and subsequent extubation were assessed using the Oral Motor Function (OMF) subscore. As a secondary, exploratory analysis, we included non-stroke patients to test the applicability of the OMF subscore beyond the stroke population. As the DEFISS is specific to stroke-related risk factors, it was not applied in this group. The following analyses are therefore exploratory and limited to OMF reliability, correlation with FEES-based FEDSS, and association with extubation outcomes.

## Methods (exploratory analysis)

OMF ratings were obtained by three independent raters (novice, intermediate, expert). Interrater reliability was assessed using intraclass correlation coefficient (ICC, two-way mixed-effects, absolute agreement). Construct validity was explored by Spearman correlation between OMF and FEDSS after extubation. An exploratory dichotomization of OMF scores (≥4 vs. <4) was tested against extubation failure using Fisher’s exact test.

## Results

Patient characteristics are summarized in Table S1. Interrater reliability of OMF was excellent (ICC = 0.921, 95% CI 0.782–0.977). OMF correlated positively, though not significantly, with FEDSS (Spearman’s ρ = 0.38, p = 0.26, n = 11). Sensitivity analyses included (A) novice-only and (B) averaged-rater thresholds (Table S2). Exploratively, patients with OMF <4 experienced higher rates of extubation failure (Table S4). Given the small sample size, these findings should be considered hypothesis-generating only.

**Table S1. Baseline characteristics of non-stroke patients (n = 11).**

| Characteristic | Value |
| --- | --- |
| Age, mean ± SD (years) | 70.45 ± 9.15 |
| Female sex, n (%) | 5 (45.5%) |
| Primary diagnosis, n (%) |  |
| Seizure/Status epilepticus | 5 (45.5%) |
| Meningitis/Encephalitis | 4 (36.4%) |
| Other | 2 (18.2%) |
| Duration of mechanical ventilation, mean ± SD (h) | 124.55 ± 83.98 |
| Reintubation, n (%) | 3 (27.3%) |
| due to dysphagia, n (%) | 3 (27.3%) |
| Pneumonia after extubation, n (%) | 4 (36.4%) |
| Tracheostomy during admission, n (%) | 3 (27.3%) |
| In-hospital mortality, n (%) | 0 (0%) |

**Table S2. Interrater reliability of OMF in non-stroke patients.**

| Score | ICC (95% CI) |
| --- | --- |
| OMF total score (average measures) | 0.921 (0.782 – 0.977) |
| OMF total score | 0.795 (0.544 – 0.934) |

# Table S3. ROC coordinates for DEFISS predicting dysphagia-related reintubation

n = 39, events = 5. Youden’s J = Sensitivity + Specificity – 1. The optimal cutoff (highest J) is highlighted in bold.

| Cutoff (≥) | Sensitivity | 1 – Specificity | Specificity | Youden’s J |
| --- | --- | --- | --- | --- |
| –0.67 | 1.00 | 1.000 | 0.00 | 0.000 |
| 0.67 | 1.00 | 0.971 | 0.03 | 0.029 |
| 1.33 | 0.80 | 0.676 | 0.32 | 0.124 |
| 1.83 | 0.80 | 0.647 | 0.35 | 0.153 |
| 2.17 | 0.80 | 0.471 | 0.53 | 0.329 |
| 2.50 | 0.80 | 0.441 | 0.56 | 0.359 |
| 2.83 | 0.80 | 0.382 | 0.62 | 0.418 |
| **3.33** | ****0.80**** | ****0.294**** | ****0.71**** | ****0.506**** |
| 3.83 | 0.60 | 0.265 | 0.74 | 0.335 |
| 4.17 | 0.40 | 0.088 | 0.91 | 0.312 |
| 4.67 | 0.40 | 0.029 | 0.97 | 0.371 |
| 5.17 | 0.40 | 0.000 | 1.00 | 0.400 |
| 6.17 | 0.20 | 0.000 | 1.00 | 0.200 |
| 8.00 | 0.00 | 0.000 | 1.00 | 0.000 |

Note. The Youden-optimal cutoff was DEFISS ≥ 3.33 (J = 0.506; sensitivity 0.80; specificity 0.71). The pre-specified threshold remained DEFISS ≥ 4. SPSS flagged ties; upper CI truncated at 1.000 in the main text.

**Table S4. Association of OMF ≥4 with extubation failure (non-stroke patients).**

| OMF group | Extubation failure | Extubation success |
| --- | --- | --- |
| OMF <4 | 3 | 6 |
| OMF ≥4 | 0 | 2 |

Abbreviations: OMF = Oral Motor Function; FEDSS = Fiberoptic Endoscopic Dysphagia Severity Scale; ICC = Intraclass Correlation Coefficient.

**Oral Motor Function Scale** (adapted from Colonel et al. 2008^35^)

| OMF | Saliva  Management | Swallowing | Lip Closure | Tongue Motility | Jaw Motility |
| --- | --- | --- | --- | --- | --- |
| 0 | No pooling | Spontaneous | Complete | Regular | Regular |
| 1 | Pooling in pharynx | After stimulation | Incomplete | Limited | Limited |
| 2 | Pooling in mouth | None | Atone | Atone | Atone |
| 3 | Anterior leakage | … | … | … | … |

**Determine Extubation Failure in Severe Stroke** (adapted from Suntrup-Krueger et al. 2019^15^)

| Item | Points |
| --- | --- |
| Duration of ventilation (h)  < 24  ≥ 24 | **0**  **1** |
| Examination of oral motor function (OMF, pts)  < 4  ≥ 4 | **0**  **2** |
| Infratentorial lesion  No  Yes | **0**  **2** |
| Stroke Severity prior to extubation (NIHSS, pts)  < 5  5 – 15  > 15 | **0**  **1**  **2** |
