# Supplementary material for: Reliability and validity of the DEFISS score for predicting post-extubation dysphagia and dysphagia-related reintubation after stroke
Source: Sci Rep. 2026 Jul 17;16:22536. doi: 10.1038/s41598-026-62133-x (PMC13379578; doi:10.1038/s41598-026-62133-x)
Supplement: Supplementary file 2 — Supplementary Material 2 [file 41598_2026_62133_MOESM2_ESM.docx]

**STROBE and GRRAS Checklists**

**Manuscript title:**

Interrater reliability and validity of the Determine Extubation Failure in Severe Stroke (DEFISS) score for predicting post-extubation dysphagia and dysphagia-related reintubation after stroke

**Journal:**

*Neurological Research and Practice (NRP)*

**Corresponding author:**

Paul Muhle, MD

Department of Neurology, University Hospital Münster

Albert-Schweitzer-Campus 1, 48149 Münster, Germany

Email: paul.muhle@ukmuenster.de

**Date:** October 2025

**STROBE Checklist – Observational Cohort Study**

**1. Title and abstract** – Title includes study design; abstract specifies prospective observational cohort and reliability/validity objectives.

**2. Background/rationale** – Introduction, paragraphs 1–3: Rationale and clinical relevance of post-extubation dysphagia after stroke.

**3. Objectives** – Introduction, final paragraph: Aim to evaluate interrater reliability and validity of DEFISS score.

**4. Study design** – Methods, Design and setting: Prospective observational design at tertiary neuro-ICU.

**5. Setting** – Methods, Design and setting: single-center, University Hospital Münster, Germany.

**6. Participants** – Methods: Inclusion criteria (stroke, planned extubation), exclusion (non-stroke exploratory).

**7. Variables** – Methods: DEFISS components and FEES outcomes described in detail.

**8. Data sources/measurement** – Methods: DEFISS scored by three raters; FEES performed post-extubation; FEDSS as gold-standard reference.

**9. Bias** – Blinding of raters and assessors; prospective design to minimize selection bias.

**10. Study size** – All eligible stroke patients between May 2023 and August 2025; n = 39 stroke cases.

**11. Quantitative variables** – DEFISS and OMF as continuous/ordinal; FEDSS ordinal; reintubation binary.

**12. Statistical methods** – Methods, Statistical analysis: ICCs, ROC AUC, Spearman correlation, 2×2 metrics, Brier score.

**13. Participants (Results)** – Results, Cohort: 39 included, patient flow in Figure 1.

**14. Descriptive data** – Table 1: Demographic and clinical characteristics.

**15. Outcome data** – Results, Predictive validity: dysphagia-related reintubation, n = 5/39 (12.8%).

**16. Main results** – Table 4 and text: ICCs, OR, sensitivity/specificity, AUC 0.74.

**17. Other analyses** – Supplemental Tables S1–S3: sensitivity analyses (novice-only, averaged-rater).

**18. Key results** – Discussion, paragraph 1: Summary of main findings and clinical meaning.

**19. Limitations** – Discussion, Limitations and future work: single-center design, low event rate, generalizability.

**20. Interpretation** – Discussion: clinical implications, comparison with existing tools, integration with FEES workflow.

**21. Generalizability** – Discussed under limitations; need for external multicenter validation.

**GRRAS Checklist – Reliability and Agreement Studies**

**1. Identification** – Title and abstract clearly identify reliability and agreement study.

**2. Background** – Introduction provides rationale for assessing interrater reliability of DEFISS and OMF.

**3. Objectives** – Explicitly stated: evaluate interrater reliability and validity of DEFISS in stroke.

**4. Study design** – Prospective, blinded, three-rater design described in Methods.

**5. Sample size** – n = 39 stroke patients; justified by consecutive sampling and feasibility.

**6. Participants** – All stroke patients meeting inclusion criteria; non-stroke analyzed exploratively.

**7. Raters** – Three raters (novice, intermediate, expert) defined by ICU experience strata.

**8. Test method** – DEFISS scoring at bedside within 3 hours before planned extubation.

**9. Statistical analysis** – Two-way mixed ICCs (absolute agreement) for total and subscore; Cohen’s κ and Fleiss’ κ for binary cutoff (DEFISS ≥4).

**10. Precision** – Reported 95% CIs for ICCs, AUCs, and ORs.

**11. Results** – Reliability results in Tables 2–3; validity metrics in Table 4.

**12. Interpretation** – Discussion: excellent reliability, moderate predictive validity, and clinical implications.

**13. Limitations** – Low event count and single-center design acknowledged.

**14. Generalizability** – Recommendation for external multicenter validation.
